# Maternal Smoking during Pregnancy and Daughters’ Preeclampsia Risk

**DOI:** 10.1371/journal.pone.0144207

**Published:** 2015-12-02

**Authors:** Kristina Mattsson, Karin Källén, Anna Rignell-Hydbom, Stefan R. Hansson, Thomas F. McElrath, David E. Cantonwine, Lars Rylander

**Affiliations:** 1 Division of Occupational and Environmental Medicine, Institute of Laboratory Medicine, Lund University, Lund, Sweden; 2 Department of Obstetrics and Gynecology, Institute for Clinical Sciences, Lund University, Lund, Sweden; 3 Division of Maternal-Fetal Medicine, Department of Obstetrics and Gynecology, Brigham and Women’s Hospital, Harvard Medical School, Boston, Massachusetts, United States of America; University of Missouri, UNITED STATES

## Abstract

**Background:**

An obstetrical paradox is that maternal smoking is protective for the development of preeclampsia. However, there are no prior studies investigating the risk of preeclampsia in women who were exposed to tobacco smoking during their own fetal period. We aimed to study the subsequent risk of preeclampsia in women who were exposed to tobacco smoke *in utero*, using a national population-based register.

**Methods:**

Data were obtained from the Medical Birth Register of Sweden for women who were born in 1982 (smoking data first recorded) or after, who had given birth to at least one child; 153 885 pregnancies were included.

**Results:**

The associations between intrauterine smoking exposure (three categories: non-smokers, 1–9 cigarettes/day [moderate exposure], and >9 cigarettes/day [heavy exposure]) and subsequent preeclampsia (n = 5721) were assessed using logistic regressions. In models adjusted for maternal age, parity and own smoking, the odds ratios (OR) for preeclampsia were 1.06 [95% CI: 0.99,1.13 for moderate intrauterine exposure, and 1.18, [95% CI: 1.10,1.27] for heavy exposure. Estimates were slightly strengthened in non-smoking women who experienced heavy intrauterine exposure (adjusted OR 1.24 [95% CI: 1.14,1.34]). Results were no longer statistically significant after adjustment for the woman’s own BMI, gestational age and birthweight Z-scores.

**Conclusion:**

These data revealed some evidence of a possible weak positive association between intrauterine smoking exposure and the risk of subsequent preeclampsia, however, results were not significant over all manifestations of preeclampsia and confounder adjustment. The increased risk might be mediated through exposed women’s own BMI or birthweight.

## Introduction

Preeclampsia and other hypertensive disorders during pregnancy constitute a major cause of maternal and perinatal morbidity worldwide.[1] In spite of preeclampsia being a significant clinical problem, affecting between 3–7% of pregnancies in industrialized countries, much remains unclear regarding the etiology of this condition.[2]

Preeclampsia is generally considered to develop in two stages.[3, 4] The first stage is characterized by defects in implantation and formation of the placenta. As a result of an impaired blood perfusion, oxidative stress causes release of several factors that leak into the maternal circulation, where they cause inflammation and general endothelial damage, that eventually give rise to the clinical manifestations seen in stage two.[2, 4]

There is a robust body of literature that consistently has demonstrated a reduced risk of preeclampsia in women who smoke when they are pregnant.[5–7] However, when preeclampsia develops in smoking women, it is often a more severe form (earlier onset, greater hypertension and/or greater proteinuria),[7] although this finding is not replicated in all studies.[8] Adverse perinatal outcomes related to preeclampsia have been shown to be more prevalent in smoking women as compared to non-smoking women with preeclampsia,[5] however, there are also studies that report a lower risk for adverse perinatal outcomes when preeclamptic women smoke.[9, 10]

The relationship between tobacco products and preeclampsia has been made more complex by recent findings in women exposed to environmental tobacco smoke as well as previous smokers, where one study showed no association with preeclampsia,[11] and another reported an increased risk following these exposures.[12] One Swedish register study also found that when women quit smoking sometime between the first antenatal visit (at 8–12 weeks of gestation) and the third trimester visit (at 30–32 weeks), the reduced preeclampsia risk was no longer apparent.[7] Among women using snuff (smokeless tobacco) during their pregnancy, there are also conflicting results as to its association with preeclampsia.[7, 13]

No earlier study has examined the relationship between exposure to tobacco smoking *in utero* and later risk of developing preeclampsia. This could be relevant, as intrauterine smoking exposure repeatedly has been established as a risk factor for several adverse perinatal outcomes, including intrauterine growth restriction and preterm birth, which in turn have been linked to a higher risk of developing preeclampsia as well as pregnancy-induced hypertension in several studies.[14–19] Considering the two-stage model, both placental and maternal factors are relevant for the development and clinical manifestation of preeclampsia, and the condition is associated with low-grade systemic inflammation analogous to the inflammation seen in adults with hypertension, obesity and diabetes.[4] Rather than having an effect on implantation or placental morphology, we hypothesized that there could be a link between intrauterine smoking exposure and later preeclampsia via modification of maternal constitutional factors, resulting in an increased susceptibility, or less compensatory ability, to substances released by the preeclamptic placenta.

The aim of this study was therefore to investigate the risk of preeclampsia following smoking exposure during the woman’s own fetal life. We intended to examine the possible relation between *in utero* tobacco exposure and both the gestational age of onset, as well as the clinical severity, of preeclampsia in later life.

## Methods

### Data selection

The data for this study come from the Medical Birth Register (MBR) of Sweden. The register, established in 1973, covers around 97–99% of all births in Sweden.[20] Data are collected during the free antenatal visits offered to all women in Sweden, as well as around birth of the child. Out of the approximate ten antenatal visits, which is recommended if the pregnancy is without complications, two contain lengthier interviews from which data are reported to the National Board of Health and Welfare. Data reporting is mandatory, and no informed consent is required. The first visit typically occurs at 8–12 weeks of gestation. In 1982, the register began recording information on smoking behavior during pregnancy, hence, the cohort used here consists of women who were born in 1982 or later, and who have subsequently given birth to at least one child. Data were retrieved through 2013. All individuals in Sweden are assigned a 10-digit personal identification number at birth, and the MBR records both the mother’s and infant’s number at delivery. Therefore, linkages between generations are possible within the register. Of a total of 195,922 eligible pregnancies, those with missing data on smoking behavior in either generation 1 (G1) or generation 2 (G2) were excluded, leaving 161,458 pregnancies (82.4%). Some of the G2 women in the cohort contributed with more than one pregnancy, n = 49,893 (32.3%).

### Exposure assessment

Pregnant women in both generations were interviewed by trained midwives using standardized questionnaires and reported their current smoking behavior during their first antenatal visit. This is categorized in the MBR as: non-smoker, 1–9 cigarettes/day or >9 cigarettes/day. There are data on maternal smoking during the last trimester collected at around 30–32 weeks of gestation. However, this variable was added later and the large amount of missing data (98.1% missing for G1 women) precluded the use of this variable in the present study.

### Definition of outcomes

Earlier evidence points towards a heterogeneity of both risk factors and clinical manifestations of preeclampsia.[4, 21–23] Trying to account for this, we wanted to investigate G2 women’s risk of preeclampsia, both in terms of clinical severity of the disease based on diagnosis coding, as well as in terms of timing of onset (i.e. early- or late-onset).

MBR registers any pregnancy-related diagnoses using the Swedish version of the International Classification of Diseases (ICD). During the time frame studied here, the eighth revision (ICD-8) was used until 1986, the ninth revision (ICD-9) from 1987 until 1996, and the tenth revision (ICD-10) since 1997 and onwards.

Throughout Sweden, preeclampsia is defined as at least two measurements of a diastolic blood pressure of ≥90 mmHg, in combination with proteinuria (≥0.3 g/day or ≥1+ on a urine dipstick). A division is made into mild and severe, where the former (ICD-8: 637.03, ICD-9: 642E and ICD-10: O14.0) is defined as a diastolic blood pressure of 90–109 mmHg combined with proteinuria of <5 g/day or 1+ or 2+ on a urine dipstick, and the latter (ICD-8: 637.04 and 637.10; ICD-9: 642F and 642G; and ICD-10: O14.1 and O15) is defined as either a diastolic blood pressure of ≥110 mmHg or proteinuria of ≥5 g/day, or a combination of both.

Prior classifications include preeclampsia non–ultra descriptus (ICD-8: 637.09, ICD-9: 642H and ICD-10: O14.9) and toxicosis (ICD-8: 637.99). These categories were both considered as mild preeclampsia in the present study.

Generally, a distinction is made between early-onset preeclampsia (before week 34) and late-onset (from week 34 and onwards), where early-onset preeclampsia is associated with more placental pathology, as well as higher maternal and fetal morbidity, whereas maternal factors are considered more important for late-onset preeclampsia.[21, 23, 24] MBR does not record date of diagnosis, making the distinction difficult in the present setting. However, it is clinical practice in Sweden that any woman with an early-onset preeclampsia is admitted to hospital for evaluation, and according to international guidelines, induction of labor is recommended pre-term. Therefore, registered combination of a preeclampsia diagnosis and pre-term delivery before week 34, could constitute a proxy for early-onset preeclampsia. For all analyses on early-onset preeclampsia, this definition was used.

### Covariates

**Fig 1:**
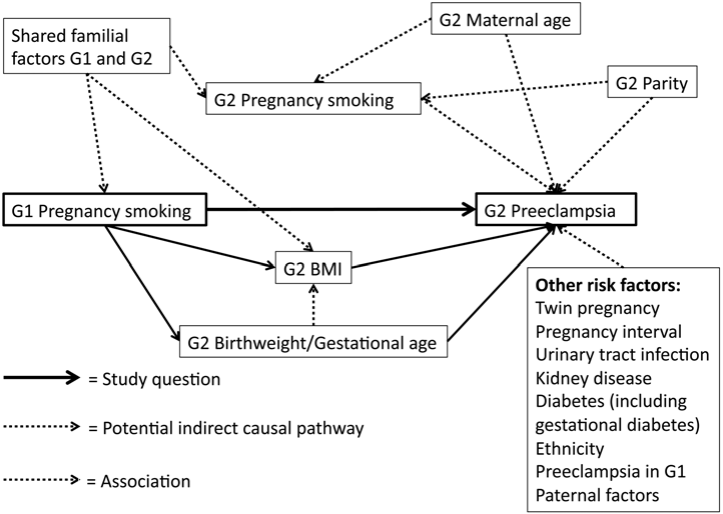
Conceptual frame-work regarding potential confounders and intermediates for the association between prenatal smoking exposure and risk of preeclampsia. Figure notes: G1—first generation G2—second generation

Other covariates obtained from the MBR were: (G2) woman’s age at pregnancy (three categories: <20, 20–29, and 30–39 years), parity (three categories: 1, 2, and ≥3), own smoking in early pregnancy (three categories: non-smoker, 1–9 cigarettes/day, and >9 cigarettes/day), BMI calculated from height and early pregnancy weight measured at first antenatal visit, (six categories: <18.5 18.5–24.9, 25–29.9, 30–34.9, 35–39.9 and ≥40 kg/m^2^), G2 own birthweight Z-scores (continuous), and own gestational age (five categories: <32, 32–34, 35–36, 37–42 and >42 completed weeks of gestation). A conceptual model depicting how we have considered potential confounders and intermediates in relation to the exposure and outcome is shown in Fig 1.

In all the following analyses, only G2 women with valid data on BMI (missing n = 6,238 [3,9%]) were included. An additional n = 1,255 (0.8%) were excluded due to missing birthweight Z-scores, n = 27 due to missing data on parity and n = 53 on country of birth, respectively, leaving a total n = 153,885 with valid data on all covariates.

### Statistical analyses

The association between intrauterine smoking exposure and subsequent preeclampsia in G2 women was assessed through logistic regressions generating odds ratios (OR) with 95% confidence intervals (CI).

Initial models were adjusted for woman’s (G2) age at pregnancy, parity and own smoking in early pregnancy. As maternal BMI is a risk factor for preeclampsia, and also has been shown in some studies to be a possible effect of intrauterine smoking exposure,[25, 26] BMI could potentially act as a mediator in the causal pathway (Fig 1). In that case, it should not be included as a confounder. The same argument could be made for the woman’s birthweight and gestational age. We thus report analyses that included maternal BMI, and additionally gestational age and birthweight Z-scores, as separate models.

Because there is a possible interaction between intrauterine smoking exposure (G1 smoking) and woman’s own smoking in pregnancy (G2 smoking), we also considered this. An interaction was considered important if the p-value for the interaction term was below 0.05. If so, separate analyses were performed among G2 women who smoked in pregnancy and among G2 women who did not.

It has been reported previously that fetal sex might influence risk and severity of preeclampsia both in singleton and twin pregnancies; it is more common to develop preeclampsia if carrying a male fetus, but women have a higher risk to develop a more severe form early in gestation, with worse perinatal outcomes, if carrying a female fetus.[27–29] Since this sexual dimorphism shows some similarities with early- versus late-onset preeclampsia, we wanted to test if stratifying by fetal sex in the G2 pregnancy additionally supported our hypothesis that prenatal smoking exposure might influence later preeclampsia risk via an effect on the maternal response.

To address a possible impact of heredity, we conducted separate analyses where we excluded any subject whose mother (G1) developed preeclampsia during her pregnancy.

Further, the following sensitivity analyses to evaluate the robustness of the results were performed: i) including only primiparous G2 women, ii) including only singleton pregnancies, iii) including only Swedish-born G1 women, and iv) excluding any G2 women with previous hypertension (chronic or pregnancy-induced). To account for clustering of data (G2 sisters within the same G1 mother), we also performed all analyses including only independent G2 mothers giving birth for the first time.

All analyses were carried out using SPSS version 21.0 (SPSS Inc., Chicago, IL, USA).

## Results

This study covers all births in Sweden between 1997 and 2013, by women born 1982 or later. Table 1 shows selected perinatal characteristics in the G2 generation by their exposure to G1 pregnancy smoking. G2 women who were exposed to smoking *in utero* were younger, had higher BMI and smoked more often when they gave birth themselves, compared to non-exposed mothers (p for all covariates < 0.001). The G2 generation was younger at childbirth (age range 13–30 years), and more likely to be primiparous compared to G1 (S1 Table). There was also a higher prevalence of overweight mothers in G2. The prevalence of preeclampsia in G2 was 3.7%. Among the mothers with early-onset preeclampsia, 45% also had a diagnosis code denoting severe preeclampsia. In 21.3% of the preeclamptic G2 mothers, preeclampsia was seen in combination with a child being small-for-gestational age.

**Table 1:** Characteristics in the second generation (G2) pregnancy by first generation (G1) smoking. Numbers are shown in n and (%).

	*G1 Non-smoking*	*G1 Smoking*
Women total	n = 95,763	n = 58,122
**Age at childbirth (yrs)**		
<20	6,339 (6.6)	5,837 (10.0)
20–29	88,649 (92.6)	51,997 (89.5)
30–39	775 (0.8)	288 (0.5)
**Body mass index (kg/m** ^**2**^ **)**		
<18.5	3,389 (3.5)	1,838 (3.2)
18.5–24.9	59,043 (61.7)	31,386 (54.0)
25–29.9	22,112 (23.1)	15,015 (25.8)
30–34.9	7,907 (8.3)	6,549 (11.3)
35–39.9	2,419 (2.5)	2,399 (4.1)
≥40	893 (0.9)	935 (1.6)
**Parity**		
1	65,904 (68.8)	38,088 (65.5)
2	25,425 (26.5)	16,454 (28.3)
≥3	4,434 (4.6)	3,580 (6.2)
**Number in birth**		
Singleton	93,962 (98.1)	56,952 (98.0)
Twins/multiple	1,801 (1.9)	1,170 (2.0)
**Smoking during pregnancy**		
Non-smoker	88,214 (92.1)	45,086 (77.6)
1–9 cigarettes/day	6,359 (6.6)	10,229 (17.6)
>9 cigarettes/day	1,190 (1.2)	2,807 (4.8)
**Preeclampsia** ^a^		
Mild	2,458 (2.6)	1,535 (2.6)
Severe	1,075 (1.1)	653 (1.1)
**Hypertension**		
Chronic	165 (0.2)	95 (0.2)
Pregnancy-induced	1,091 (1.1)	682 (1.2)
**Gestational diabetes**	445 (0.5)	440 (0.8)
**Non-gestational diabetes** ^b^	610 (0.6)	309 (0.5)

^a^Mild preeclampsia was defined as a diastolic blood pressure of 90–109 mmHg combined with proteinuria of <5 g/day and severe preeclampsia as either a diastolic blood pressure of ≥110 mmHg or proteinuria of ≥5 g/day or both.

^b^Includes both type 1 and type 2 diabetes as the Swedish Medical Birth Register does not distinguish between the two types.

G1 women with missing data on smoking during pregnancy were similar to those with complete smoking data regarding perinatal characteristics (S2 Table); for G2, a high proportion of those with missing smoking data also had missing data on BMI.

For moderate intrauterine smoking exposure, there was no evidence of an association with preeclampsia risk, except for late-onset preeclampsia (Table 2). In the heavily exposed group, the adjusted odds ratio (aOR) was 1.18 (95% CI: 1.10–1.27) for any type of preeclampsia. The association was attenuated and lost statistical significance when G2 BMI and own gestational age and birthweight Z-scores were added to the models (Table 2). For late-onset preeclampsia, odds ratios remained significant also when BMI was adjusted for (aOR = 1.08 [95% CI: 1.00, 1.18]).

**Table 2:** Odds ratios (OR) with 95% confidence intervals (CI) for the associations between maternal smoking during pregnancy and daughters’ risk of preeclampsia.

	*Cases*	*Non-cases*	*Crude OR (95% CI)*	*Adjusted* ^a^ *(without BMI) OR (95% CI)*	*Adjusted* ^b^ *(with BMI) OR (95% CI)*	*Adjusted* ^c^ *(with birthweight and gestational age) OR (95% CI)*
Women total (n = 153885)						
**Preeclampsia (any)**						
Non-smokers	3,533	92,230	1.00 (reference)	1.00 (reference)	1.00 (reference)	1.00 (reference)
1–9 cigarettes/day	1,240	32,759	0.99 (0.93, 1.06)	1.06 (0.99, 1.13)	0.98 (0.92, 1.05)	0.93 (0.87, 0.99)
>9 cigarettes/day	948	23175	1.07 (0.99, 1.15)	1.18 (1.10, 1.27)	1.07 (0.99, 1.15)	0.99 (0.92, 1.07)
**Preeclampsia (mild)** ^d^						
Non-smokers	2,258	93,305	1.00 (reference)	1.00 (reference)	1.00 (reference)	1.00 (reference)
1–9 cigarettes/day	875	33,124	1.00 (0.93, 1.08)	1.07 (0.99, 1.16)	0.99 (0.92, 1.07)	0.94 (0.87, 1.02)
>9 cigarettes/day	660	23,463	1.07 (0.98, 1.17)	1.18 (1.08, 1.29)	1.05 (0.96, 1.15)	0.99 (0.90, 1.08)
**Preeclampsia (severe)** ^d^						
Non-smokers	1,075	92,230	1.00 (reference)	1.00 (reference)	1.00 (reference)	1.00 (reference)
1–9 cigarettes/day	365	32,759	0.96 (0.85, 1.08)	1.03 (0.91, 1.16)	0.97 (0.85, 1.09)	0.90 (0.79, 1.01)
>9 cigarettes/day	288	23,175	1.07 (0.94, 1.22)	1.19 (1.04, 1.36)	1.09 (0.95, 1.25)	0.99 (0.87, 1.14)
**Preeclampsia (early onset)** ^e^						
Non-smokers	814	94,949	1.00 (reference)	1.00 (reference)	1.00 (reference)	1.00 (reference)
1–9 cigarettes/day	258	33,741	0.89 (0.78, 1.03)	0.95 (0.82, 1.09)	0.88 (0.76, 1.02)	0.83 (0.72, 0.96)
>9 cigarettes/day	209	23,914	1.02 (0.88, 1.19)	1.11 (0.95, 1.30)	1.00 (0.86, 1.17)	0.93 (0.79, 1.09)
**Preeclampsia (late onset)**						
Non-smokers	2719	92,230	1.00 (reference)	1.00 (reference)	1.00 (reference)	1.00 (reference)
1–9 cigarettes/day	982	32,759	1.02 (0.94, 1.10)	1.09 (1.01, 1.17)	1.01 (0.94, 1.09)	0.96 (0.89, 1.03)
>9 cigarettes/day	739	23,175	1.08 (0.99, 1.18)	1.20 (1.10, 1.31)	1.08 (1.00, 1.18)	1.01 (0.93, 1.10)

^a^Model adjusted for woman’s (G2) age at childbirth, parity, and own smoking during early pregnancy.

^b^Model adjusted for G2 age at childbirth, parity, own smoking during early pregnancy and BMI in early pregnancy.

^c^Model adjusted for G2 age at childbirth, parity, own smoking during early pregnancy, BMI and own gestational age and own birthweight Z-scores.

^d^Mild preeclampsia was defined as a diastolic blood pressure of 90–109 mmHg combined with proteinuria of <5 g/day and severe preeclampsia as either a diastolic blood pressure of ≥110 mmHg or proteinuria of ≥5 g/day or both.

^e^Presence of preeclampsia and delivery of a child before gestational week 34 used as a proxy for early-onset preeclampsia.

There was an interaction between G1 and G2 smoking during pregnancy (p<0.05). Stratified analyses according to smoking behavior in G2 are shown in Table 3. In non-smoking G2 women, point estimates were strengthened (aOR = 1.24 [95% CI: 1.14, 1.34]) and there were significant positive associations for heavy intrauterine smoking exposure with preeclampsia through all confounder adjustment except when gestational age and birthweight Z-scores were included (Table 3).

**Table 3:** Odds ratios (OR) with 95% confidence intervals (CI) for preeclampsia by exposure to intrauterine tobacco smoking, stratified according to own smoking in generation 2 (G2).

	*G2 Non-smokers (n = 133*,*300)*					
	*Cases*	*Non-cases*	*Crude OR (95% CI)*	*Adjusted* ^a^ *(without BMI) OR (95% CI)*	*Adjusted* ^b^ *(with BMI) OR (95% CI)*	*Adjusted* ^c^ *(with birthweight and gestational age) OR (95% CI)*
**Preeclampsia (any)**						
Non-smokers	3,313	84,901	1.00 (reference)	1.00 (reference)	1.00 (reference)	1.00 (reference)
1–9 cigarettes/day	1,070	26,174	1.05 (0.98, 1.12)	1.07 (1.00, 1.15)	0.99 (0.93, 1.07)	0.94 (0.87, 1.01)
>9 cigarettes/day	797	17,045	1.20 (1.11, 1.30)	1.24 (1.14, 1.34)	1.10 (1.02, 1.20)	1.03 (0.95, 1.12)
	*G2 Smokers (n = 20*,*585)* ^d^					
	*Cases*	*Non-cases*	*Crude OR (95% CI)*	*Adjusted* ^a^ *(without BMI) OR (95% CI)*	*Adjusted* ^b^ *OR (with BMI) OR (95% CI)*	*Adjusted* ^c^ *OR (with birthweight and gestational age) OR (95% CI)*
**Preeclampsia (any)**						
Non-smokers	220	7,329	1.00 (reference)	1.00 (reference)	1.00 (reference)	1.00 (reference)
1–9 cigarettes/day	170	6,585	0.86 (0.70, 1.05)	0.87 (0.71, 1.06)	0.85 (0.69, 1.04)	0.78 (0.63, 0.96)
>9 cigarettes/day	151	6,130	0.82 (0.67, 1.01)	0.84 (0.68, 1.03)	0.81 (0.66, 1.00)	0.74 (0.59, 0.91)

^a^Model adjusted for woman’s (G2) age at childbirth and parity.

^b^Model adjusted for G2 age at childbirth, parity and BMI in early pregnancy.

^c^Model adjusted for G2 age at childbirth, parity, BMI and own gestational age and own birthweight Z-scores.

^d^ ≥ 1 cigarettes/day.

When stratifying the analyses according to fetal sex, odds ratios remained unchanged when only women carrying female fetuses were included, compared to both sexes analyzed together, but were somewhat higher when the corresponding analyses were made for male fetuses (OR = 1.13 [95% CI: 1.03,1.25] and aOR = 1.25 [95% CI: 1.13,1.38] in the heavily exposed category.)

Excluding G1 women with preeclampsia marginally strengthened the results (data not shown). When including only primiparous G2 women, only singleton pregnancies, only Swedish-born G1 women, and when excluding G2 women with previous chronic or pregnancy-induced hypertension, the results remained unchanged through all analyses (data not shown).

When including only independent women in the G2 generation giving birth for the first time (n = 103,387), estimates did not change in any of the analyses (data not shown).

## Discussion

Our analyses provided some evidence of a potential weak positive association between prenatal smoking exposure and developing some manifestations of preeclampsia, however, this association was not significant over all confounder adjustments. The associations were most apparent when considering G2 non-smoking women who were exposed to >9 cigarettes/day during their fetal life, and for late-onset preeclampsia. Including women’s BMI, birthweight or gestational age in the statistical models resulted in risk estimates loosing statistical significance, suggesting that these variables could potentially mediate some of the associations.

This is the first study investigating intrauterine smoking exposure and later preeclampsia risk. Maternal smoking during pregnancy is known to increase the risk of low birthweight and preterm birth, which in turn have been found in several studies to be associated with preeclampsia or gestational hypertension.[14–19] Considering this, we hypothesized that there might be an association between smoking exposure *in utero* and preeclampsia risk, either working along similar pathophysiological pathways as the association with birthweight, or independently. We supposed that an effect would be mainly through a modification of maternal factors increasing the risk of an adverse systemic response to released placental factors reaching the maternal circulation, rather than an effect on placental implantation. We found slightly stronger estimates for late-onset preeclampsia, which was expected according to our hypothesis. This finding might reflect earlier reports regarding maternal constitutional factors being more important for late-onset preeclampsia and the second stage of disease development.[3, 21, 30] We argue that this was further supported by the finding in these data of higher risk estimates for G2 women carrying male fetuses, which has been shown to be associated with preeclampsia manifestations more similar to late-onset preeclampsia.[29]

Maternal BMI is an established important risk factor for preeclampsia, and our data indicate that it could potentially mediate the association with prenatal smoking exposure, although this variable might also have captured mediation through the woman’s birthweight. There are earlier findings indicating that intrauterine smoking exposure, independent from an effect via birthweight, increases the risk of being obese or over-weight among adult women.[25, 26] If BMI does lie on the causal pathway as an intermediate, its inclusion as a confounder in the models could be questioned, however, how to treat this variable is debatable, as noted in Fig 1. That birthweight and gestational age lie on the causal pathway seems probable, and inclusion of these covariates is not necessarily justified. However, when included, statistically significant associations were absent in all outcome models.

We found no evidence of an association with early-onset preeclampsia, consistent with our hypothesis, as this would require a more pathological placenta. A prior study, also using the Swedish Medical Birth register, examining preeclampsia risk where being born small-for-gestational age was considered as the determinant, found higher risk estimates for severe than for mild preeclampsia.[14] We did not find any notable differences in risk estimates when stratifying on severity of the disease as noted by diagnostic codes in our study. Other reports on fetal growth and subsequent preeclampsia risk have not made any distinctions as to type or severity of preeclampsia,[15, 16, 18] but some have considered other hypertensive disorders during pregnancy.[17]

Smoking during pregnancy has in previous research consistently been shown, in a dose-response manner, to reduce the risk of preeclampsia,[5–7] however not if the woman quit smoking before the last trimester.[7] Smoking women who do develop preeclampsia, however, tend to develop severe preeclampsia with worse perinatal outcomes,[5] although there are studies finding the contrary.[9, 10] One suggested explanation for the lowered risk among smokers is that the carbon monoxide in tobacco smoke lowers circulating anti-angiogenic proteins.[31] If every woman is considered to have a threshold for angiogenic imbalance, and a severe form of preeclampsia requires greater alterations in angiogenic proteins, it seems logical that smoking would be less potent in protecting those women physiologically pre-disposed for a severe form of preeclampsia, thus shifting the proportion of preeclamptic smoking women to the more severe category.[31] If carbon monoxide is a key element it could also help explain why smokeless tobacco, such as snuff, has not been seen to reduce preeclampsia risk in the same manner.[7]

The present study has some important strengths. First, all data come from a robust nation-wide register which reduces the risk of selection bias. Second, the outcome is based on clinical diagnoses, using the same diagnostic criteria over the whole country. Since practically all pregnant women in Sweden participate in the free maternal health care, and home deliveries are rare,[32] the coverage of women with preeclampsia should be relatively complete. Third, it has been previously validated that the exposure variable in this study (maternal self-report) is accurate and useful for estimating exposure during pregnancy.[33] The validation study, based on a randomly selected sub-sample from the MBR with serum measurements of cotinine taken at delivery, found a high agreement between self-reported smoking data and serum cotinine levels, as well as high correlations between maternal and umbilical cord levels.[33] Additionally, the study showed that the majority (87%) of the women who smoked in early pregnancy, also smoked at the time of delivery. This is supported by data from this cohort, where the proportion of smoking G2 women in late pregnancy (week 32) were 10.4% as compared to 13.4% at the first antenatal visit. The proportion of heavy smokers was also similar between the two visits (2.6% compared to 2.0%). Unfortunately, these numbers were only available from the G2 generation since the variable has been added later to the MBR.

However, this study has limitations. We lack information on exposures and life-style factors for a large part of these women’s lives, such as change of partner, exposure to second-hand smoking, and socio-economic status. Although there is an evident socio-economic gradient in smoking behavior, previous studies have not been able to consistently show that socio-economic status is relevant for the risk of hypertensive disorders during pregnancy.[34–36] In studies on fetal growth as a predictor of preeclampsia, no effect modification was seen by including maternal education,[15–17] and another study using maternal education as a proxy of socio-economic status found that the effect on gestational hypertension was largely mediated though maternal BMI.[37] We also lacked information on genetic factors other than a presence of preeclampsia in the G1 generation. Excluding these women, however, only marginally changed the estimates.

The proportion of women who had missing data on smoking behavior was not even between the two generations, with most missing being in G1. However, the total number of G2 women with missing smoking data was small (n = 7,607 [3.9%] of a total of 195,922 eligible pregnancies), and the prevalence of preeclampsia as well as other perinatal characteristics were very similar between the groups.

Last, the weakly increased risks presented in this study should be interpreted with caution, especially taking the lack of significance for some manifestations and the possibility of residual confounding in the present setting into account. In terms of explaining the etiology of preeclampsia in a clinical context, intrauterine smoking exposure might carry little information in the light of other risk factors.

## Conclusion

In conclusion, these data presented some evidence of a possible weak positive association between heavy intrauterine smoking exposure and the risk of subsequent late-onset preeclampsia, however, associations were not significant over all preeclampsia manifestations and confounder adjustments. The increased risk might be mediated through exposed women’s own BMI or birthweight.

## Supporting Information

S1 Table Characteristics of included women with complete smoking data in both generations. Smoking was recorded in the Medical Birth Register beginning in 1982. Numbers are shown in n and (%).
(DOCX)

S2 Table Characteristics of women in first generation (G1) and second generation (G2), according to available information on smoking during pregnancy in the Swedish Medical Birth Register. Numbers are shown in n and (%).
(DOCX)
