# Supplementary material for: Maternal Smoking during Pregnancy and Daughters’ Preeclampsia Risk
Source: PLoS One. 2015 Dec 2;10(12):e0144207. doi: 10.1371/journal.pone.0144207 (PMC4667973; doi:10.1371/journal.pone.0144207)
Supplement: S1 Table — Smoking was recorded in the Medical Birth Register beginning in 1982. Numbers are shown in n and (%). (DOCX) [file pone.0144207.s001.docx]

**S1 Table.** Characteristics of included women with complete smoking data in both generations. Smoking was recorded in the Medical Birth Register beginning in 1982. Numbers are shown in n and (%).

|  | *First generation (G1) pregnancy* | *Second generation (G2) pregnancy* |
| --- | --- | --- |
| Women total (n=153,885) |  |  |
| **Age at childbirth (yrs)** |  |  |
| <20 | 9,586 (6.2) | 12,176 (7.9) |
| 20-29 | 105,421 (68.5) | 140,646 (91.4) |
| 30-39 | 37,089 (24.1) | 1,063 (0.7) |
| ≥40 | 11,789 (1.2) | -* |
| **Body mass index (kg/m^2^)** |  |  |
| <18.5 | 9,054 (8.5) | 5,227 (3.4) |
| 18.5-24.9 | 79,252 (74.0) | 90,429 (58.8) |
| 25-29.9 | 15,323 (14.3) | 37,127 (24.1) |
| 30-34.9 | 3,055 (2.9) | 14,456 (9.4) |
| 35-39.9 | 335 (0.2) | 4,818 (3.1) |
| ≥40 | 42 | 1,828 (1.2) |
| Missing | 46,824 | - |
| **Parity** |  |  |
| 1 | 60,756 (39.5) | 103,992 (67.6) |
| 2 | 50,032 (33.8) | 41,879 (27.2) |
| ≥3 | 41,097 (26.7) | 8,014 (5.2) |
| **Country of birth** |  |  |
| Sweden | 138,670 (90.1) | 153,885 (100) |
| Other | 15,215 (9.9) | -^a^ |
| **Number in birth** |  |  |
| Singleton | 151,479 (98.4) | 150,914 (98.1) |
| Twins/multiple | 2,406 (1.6) | 2,971 (1.9) |
| **Smoking during pregnancy** |  |  |
| Non-smoker | 95,763 (62.2) | 133,300 (86.6) |
| 1-9 cigarettes/day | 33,999 (22.1) | 16,588 (10.8) |
| >9 cigarettes/day | 24,123 (15.7) | 3,997 (2.6) |
| **Preeclampsia^b^** |  |  |
| Mild | 2,162 (1.4) | 3,993 (2.6) |
| Severe | 364 (0.4) | 1,728 (1.1) |
| **Hypertension** |  |  |
| Chronic | 158 (0.1) | 260 (0.2) |
| Pregnancy-induced | 1,522 (1.0) | 1,773 (1.1) |
| **Gestational diabetes^c^** | 276 (0.2) | 885 (0.6) |
| **Non-gestational diabetes^d^** | 609 (0.4) | 919 (0.6) |

^a^No cases in dataset.

^b^Mild preeclampsia was defined as a diastolic blood pressure of 90–109 mmHg combined with proteinuria of <5 g/day and severe preeclampsia as either a diastolic blood pressure of ≥110 mmHg or proteinuria of ≥5 g/day or both.

^c^Gestational diabetes recorded since 1987. For G1, the prevalence of GDM is calculated after that year.

^d^Includes both type 1 and type 2 diabetes as the Swedish Medical Birth Register does not distinguish between the two types.
