# Supplementary material for: Maternal Smoking during Pregnancy and Daughters’ Preeclampsia Risk
Source: PLoS One. 2015 Dec 2;10(12):e0144207. doi: 10.1371/journal.pone.0144207 (PMC4667973; doi:10.1371/journal.pone.0144207)
Supplement: S2 Table — Numbers are shown in n and (%). (DOCX) [file pone.0144207.s002.docx]

**S2 Table.** Characteristics of women in first generation (G1) and second generation (G2), according to available information on smoking during pregnancy in the Swedish Medical Birth Register. Numbers are shown in n and (%).

|  | *First generation (G1)* | | *Second generation (G2)* | |
| --- | --- | --- | --- | --- |
|  | Complete smoking data  n=167,833 | Missing smoking data  n=28,089 | Complete smoking data  n=188,312 | Missing smoking data  n=7,607 |
| **Age at childbirth (yrs)** |  |  |  |  |
| <20 | 10,546 (6.3) | 1,907 (6.8) | 14,797 (7.9) | 863 (11.3) |
| 20-29 | 114,912 (68.5) | 18,623 (66.3) | 170,657 (90.6) | 6,630 (87.1) |
| 30-39 | 40,416 (24.1) | 7,200 (25.6) | 2,854 (1.5) | 113 (1.4) |
| ≥40 | 1,959 (1.2) | 359 (1.3) | -^a^ | -^a^ |
| **Body Mass Index (kg/m^2^)** |  |  |  |  |
| <18.5 | 9,849 (8.4) | 968 (8.9) | 6,072 (3.4) | 37 (3.4) |
| 18.5-24 | 86,398 (74.0) | 8,155 (74.8) | 106,686 (59.0) | 630 (58.7) |
| 25-29.9 | 166,90 (14.3) | 1,446 (13.3) | 43,469 (24.0) | 243 (22.6) |
| 30-34.9 | 3,351 (2.9) | 301 (2.8) | 16,930 (9.4) | 114 (10.6) |
| 35-39.9 | 368 (0.3) | 27 (0.2) | 5,618 (3.1) | 41 (3.8) |
| ≥40 | 44 | 1 | 2,127 (1.1) | 8 (0.7) |
| Total valid | 116,701 | 10,898 | 180,902 | 1,073 |
| Missing | 51,132 | 17,191 | 7,410 | 6,537 |
| **Parity** |  |  |  |  |
| 1 | 66,291 (39.5) | 11,228 (40.0) | 126,527 (67.2) | 5,059 (65.5) |
| 2 | 56,640 (33.7) | 9,774 (34.8) | 51,601 (27.4) | 2,058 (27.1) |
| ≥3 | 44,902 (26.8) | 7,087 (25.2) | 10,155 (5.4)^b^ | 490 (6.4)^b^ |
| **Mode of delivery** |  |  |  |  |
| Vaginal delivery | 149,718 (89.2) | 25,025 (89.1) | 149,744 (79.5) | 5,953 (78.2) |
| Elective Cesarean section | 715 (0.4) | 49 (0.2) | 7947 (4.2) | 367 (4.8) |
| Emergency Cesarean section | 16,813 (10.0) | 2,958 (10.5) | 16,143 (8.6) | 786 (10.3) |
| Forceps/Vacuum extraction | 587 (0.3) | 57 (0.2) | 14,478 (7.7) | 504 (6.6) |
| **Preeclampsia^c^** |  |  |  |  |
| Mild | 2,363 (1.4) | 247 (0.9) | 4,810 (2.6) | 170 (2.2) |
| Severe | 620 (0.4) | 75 (0.3) | 2,135 (1.1) | 128 (1.7) |
| **Hypertension** |  |  |  |  |
| Chronic | 175 (0.1) | 33 (0.1) | 336 (0.2) | 19 (0.2) |
| Pregnancy-induced | 1,683 (1.0) | 185 (0.7) | 2,170 (1.2) | 86 (1.1) |

^a^4 women mis-classified as >40 years in both groups.

^b^29 women had missing parity data in the group with valid smoking data, and 3 women in the group with missing smoking data, respectively.

^c^Mild preeclampsia was defined as a diastolic blood pressure of 90–109 mmHg combined with proteinuria of <5 g/day and severe preeclampsia as either a diastolic blood pressure of ≥110 mmHg or proteinuria of ≥5 g/day or both.
